# Synopsis of *Martinella* Baill. (Bignonieae, Bignoniaceae), with the description of a new species from the Atlantic Forest of Brazil

**DOI:** 10.3897/phytokeys.37.6940

**Published:** 2014-05-09

**Authors:** Alexandre R. Zuntini, Lucia G. Lohmann

**Affiliations:** 1 Departamento de Botânica, Instituto de Biociências, Universidade de São Paulo, Rua do Matão, 277, 05508-090, São Paulo, SP, Brazil

**Keywords:** *Martinella*, Bignonieae, Bignoniaceae, Neotropics, Brazilian Atlantic Forest

## Abstract

*Martinella* has traditionally included two species, *Martinella iquitoensis* and *Martinella obovata*, that are characterized by the presence of interpetiolar ridges surrounding the stems and minute prophylls of the axillary buds. A third species, *Martinella insignis*, is here described as new, illustrated and compared to other species in the genus. *Martinella insignis* is the first record of the genus in the Atlantic Forest of Brazil, and differs from other species of *Martinella* by the yellow corolla (vs. red to dark purple) and 5-lobed calices (vs. 2–4-lobed).

## Introduction

**Figure 1.**
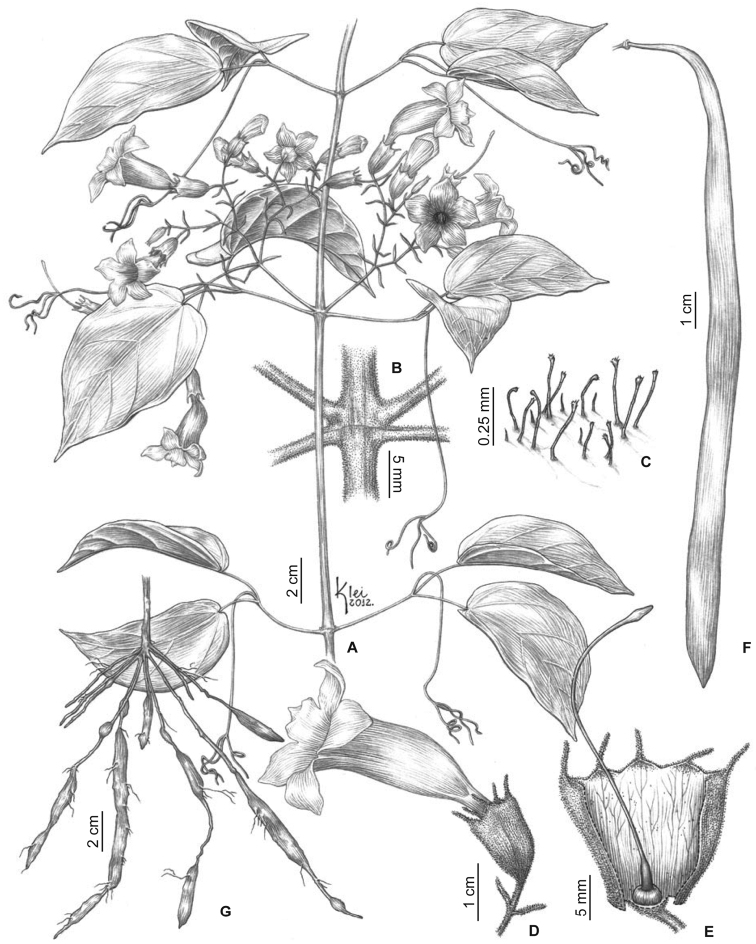
Morphology of *Martinella insignis*: **A** Habit **B** Node with interpetiolar ridge **C** Glandular stipitate trichomes **D** Flower (lateral view) **E** Calyx (opened) and gynoecium **F** Fruit **G** Root system with tuberous portions. From *Zuntini 151* (**A–E**), *Sucre 5519* (**F**) and *Zuntini 321* (**G**).

*Martinella* Baill. (1888) is strongly supported as monophyletic by molecular data (Lohmann 2006). Species of *Martinella* are well distinguished by the combination of minute triangular prophylls of the axillary buds, an interpetiolar ridge surrounding the stem, and bilobed or (2–)4-parted calyces (Lohmann and Taylor 2014); the latter two being considered putative synapomorphies of the genus (Lohmann 2006; Lohmann and Taylor 2014). In addition to these features, the basal tubular portion of the corolla is slightly longer and much narrower than the calyx, leaving it loose within the calyx, while the upper portion of the corolla tube is abruptly inflated and campanulate, up to four times wider than the tubular portion (Fig. 1D). Gentry (1974) described this corolla morphology plus the red to purple color as a Martinella-type flower, and hypothesized that this flower was associated with pollination by hummingbirds. The only exception to Gentry’s floral description is the position of the anthers (exserted or subexserted), which are always included in *Martinella*.

The genus as currently circumscribed includes two species of neotropical lianas (Lohmann and Ulloa Ulloa 2013; Lohmann and Taylor 2014): *Martinella iquitoensis* A.Samp., restricted to the Amazon basin (Brazil, Colombia, Ecuador, Peru and Venezuela) and *Martinella obovata* (Kunth) Bureau & K.Schum., ranging from Central America to Northern South America and the Amazon basin (Lohmann et al. 2013). A third morphologically distinct species of *Martinella* was discovered during fieldwork in the Atlantic Forest of Brazil; its description here extends the known range of this genus. Here we present an overview of the genus and the separation of its species.

## Materials and methods

This work is based on the study of herbarium collections of *Martinella* deposited in CEPEC, MBM, MBML, MO, NY, RB, SPF and VIES (herbarium acronyms follow Thiers 2013). Morphological descriptions are based on dried specimens, and follow the terminology of Radford et al. (1974). The parentheses in the descriptions indicate rare conditions. Micrographs of selected structures were made using a stereomicroscope and digitally processed through focus stacking. For the species distribution map, the geographical data of *Martinella insignis* were combined with those used in Lohmann et al. (2013) and plotted over a digital elevation model (GTOPO 30, available from the U.S. Geological Survey).

## Taxonomic treatment

### Martinella

Baill., Hist. Pl. 10: 30. 1888.

http://species-id.net/wiki/Martinella

#### Type.

Martinella martinii (DC.) Baill. ex K. Schum. (= *Martinella obovata* (Kunth) Bureau & K. Schum)

#### Lianas.

*Roots* with tuberous portions. *Branches* terete, glabrous or puberulous, with trichomes simple or stipitate-glandular, with continuous interpetiolar ridges, without interpetiolar glands; prophylls minute, triangular, glabrous or puberulous. *Leaves* 3-foliolate or 2-with the terminal leaflet modified into a simple or trifid tendril; leafets membranous to coriaceous, margins entire (sinuate), with or without mite-domatia, glabrous to puberulous, with glands on adaxial surface. *Inflorescences* axillary, arranged in racemes, panicles, thyrses or compound dichasia. *Flowers* with calyx tubular (campanulate), bilobed, irregularly 2–4-lobed, or 5-lobed, with lobes rounded or aristate, membranous, with few scattered glands; corolla deep purple, red or yellow, tubular in the basal portion and campanulate in the upper part, straight to weakly curved, membranous, glabrous outside, glabrous inside except with few glandular trichomes at stamen insertion; stamens included, glabrous, pollen in monads; ovary terete, smooth, glabrous or lepidote, with a single series of ovules on each placenta, style glabrous, stigma rhombic, glabrous. *Capsules* drying dark brown, linear, flattened, smooth, glabrous or puberulous, with calyx caducous; seeds oblong, winged, with wings opaque.

*Martinella* comprises three species, distributed from Mexico to eastern Brazil. The main features that distinguish the species are summarized in Table 1 and outlined in the key below.

**Table 1.** Morphological and geographical summary of *Martinella* species, based on Gentry (1977, 1982, 2009) and pers. obs.

Character	*Martinella insignis*	*Martinella iquitoensis*	*Martinella obovata*
Leaflet texture	membranous	coriaceous	membranous to coriaceous
Leaflet shape	ovate	elliptic	ovate (elliptic)
Leaflet base	cordate	cuneate	cordate to truncate (cuneate)
Leaf domatia	pocket	absent	absent
Tendril	trifid	simple (trifid)	trifid (simple)
Inflorescence	compound dichasium	thyrse or panicle	raceme
Calyx lobes	5; aristate	2–4; rounded	2–4; rounded
Corolla color	yellow	dark purple	dark purple to red (lilac)
Distribution	Eastern coast of Brazil	Amazon basin	Antilles, Central America and Northern South America through Amazon basin
Soil preference	sandy	sandy	generalist

#### Key to species of *Martinella*

**Table:** 

1	Calyx 5-lobed; corolla yellow; eastern Brazil	1. *Martinella insignis*
1’	Calyx 2–4-lobed; corolla deep wine to red; Antilles, Central America and Northern South America through Amazon basin	2
2	Inflorescence arranged in thyrse or panicle; leaflet with cuneate base	2. *Martinella iquitoensis*
2’	Inflorescence arranged in raceme; leaflet with cordate to truncate or rarely cuneate base	3. *Martinella obovata*

### 1. Martinella insignis

A.H. Gentry ex Zuntini & L.G. Lohmann sp. nov.

urn:lsid:ipni.org:names:77138471-1

http://species-id.net/wiki/Martinella_insignis

Figs 1 –2

#### Type.

BRAZIL. Bahia: Itamaraju, Rodovia Itamarajú-Teixeira de Freitas, 3km de Itamaraju (BR-101). Fazenda Chapadão, 3 November 1983, *R. Callejas, A. M de Carvalho & L. M. Silva 1629* (holotype MBM-94960!; isotypes CEPEC not seen, MO- 3600686!, NY-00483568!, RB-232556!).

#### Diagnosis.

*Martinella insignis* differs from *Martinella iquitoensis* and *Martinella obovata* by its 5-lobed calyces and yellow corollas, in contrast to 2–4-lobed calyces and dark purple to red corollas in these other species (Table 1).

#### Description.

*Lianas*. Branches green, drying brownish, striated, densely covered with stipitate-glandular trichomes when young; prophylls 0.7–1.5 × 1.0 mm, densely covered with stipitate glandular trichomes, without patelliform glands (with few patelliform glands). *Leaves* 2-foliolate with the terminal leaflet generally modified into a trifid tendril; petioles terete, 34–64 mm long, covered with stipitate glandular trichomes; petiolules terete, 14–42 mm long, covered with stipitate glandular trichomes; leaflets weakly discolorous, membranous, ovate, with a long acuminate to caudate apex and a cordate base, margins entire (sinuate), 7.6–11.8 × 3.4–6.4 cm, glabrous except on margins and main veins of the abaxial surface where stipitate glandular trichomes are found, with pocket domatia on the axils of primary and secondary veins, with few glands concentrated near base and scattered along the mid-vein on the adaxial surface. *Inflorescences* compound dichasia, with up to 7 branching orders, 9.2–12.3 cm long, sparsely to densely covered with stipitate glandular trichomes; bracts linear to narrowly elliptic, 8.4–24.7 × 0.8–3.4 mm, densely covered with stipitate glandular trichomes; pedicels terete, 5.8–19.4 mm, sparsely to densely covered with stipitate glandular trichomes. *Flowers* with calyx pale green, tubular (campanulate), 8.8–15.4 × 6.5–12 mm, sparsely covered with stipitate glandular trichomes except densely covered at the base, with few glands near the apex; lobes 5, very shallowly triangular, aristate, aristae 2.9–8.6 mm long, densely covered with stipitate glandular trichomes; corolla yellow, weakly curved, 29.5–48.3 mm long, tubular basal portion 12.7–18.9 long × 2.4–4.5 mm wide, upper campanulate portion 15.0–23.4 long × 9.2–17.2 mm wide, lobes subcircular, 3.8–9.8 × 6.1–10.9 mm, with ciliate margins; stamens in two lengths, longer ones 11.4–17.3 mm, shorter ones 12.1–15.8 mm, thecae 2.4–3.0 mm, glabrous; staminode 1.1–3.6 mm, glabrous; gynoecium 29.2–39.6 mm long; ovary glabrous; style glabrous; stigma rhomboid, glabrous; nectariferous disk 2.3–3.0 × 1.0–1.8 mm. *Capsules* 33.6–73.0 × 1.1–1.2 cm, pubescent when immature, glabrous when developed. *Seeds* ca. 1.0 × 4.6 cm.

**Figure 2.**
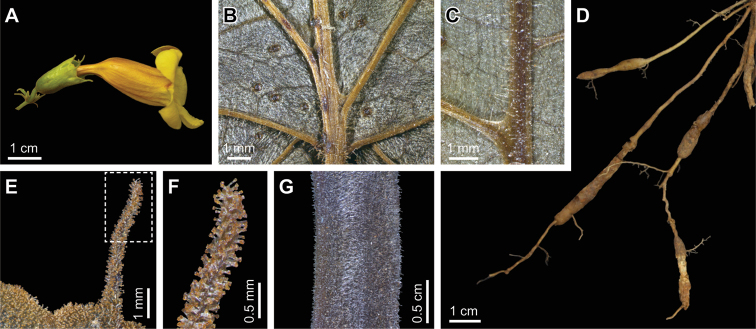
Details of *Martinella insignis*: **A** Flower **B** Leaflet base showing glands (abaxial face) **C** Mite-domatia between primary and secondary veins (abaxial side) **D** Root system with tuberous portions **E** Calyx detailing the aristae **F** Arista detail with glandular trichomes **G** Simple, tector trichomes on immature fruit. From *Zuntini 151* (**A–C, E, F**), *Zuntini 321* (**D**) and *Demuner 4481* (**G**). Micrographs were obtained using focus stacking.

#### Distribution and habitat.

**Figure 3.**
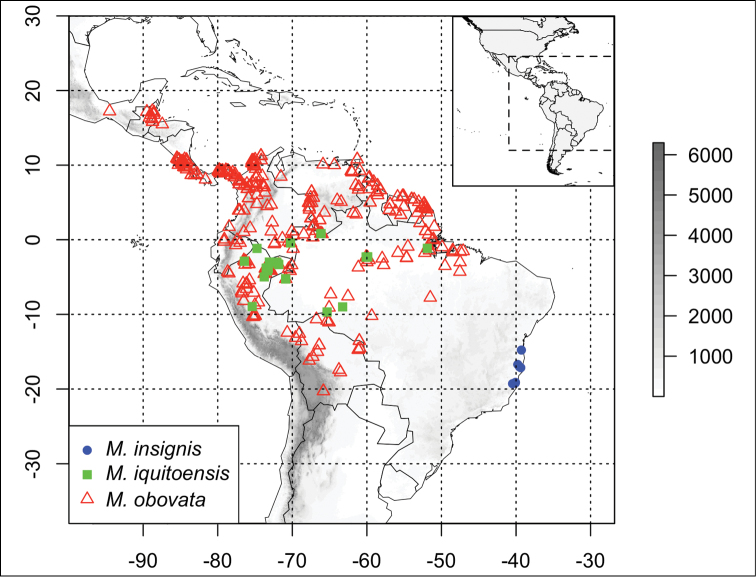
Distribution of *Martinella* species: *Martinella insignis* (solid blue circle), *Martinella iquitoensis* (solid green square) and *Martinella obovata* (open red triangles). Elevation in meters, according to the scale presented on the right.

*Martinella insignis* is restricted to the northern portion of the Brazilian Atlantic Forest, occurring predominantly in areas with sandy soils (Fig. 3).

#### Etymology.

The species epithet means remarkable or clearly distinguishable. This epithet was probably selected by Alwyn Gentry as reference to the contrasting floral color among species of *Martinella*.

#### Phenology.

Flowering specimens were collected between September and February and fruiting collections in January, September and November.

#### Conservation status.

This species is considered Data Deficient [DD] according to IUCN Standards and Petitions Subcommittee (2014) since this taxon is only known from very few specimens, with little information about its distribution and abundance. Further field studies are needed so that its conservation status can be properly documented.

#### Discussion.

*Martinella insignis* is the first species of *Martinella* found in the Atlantic Forest of Brazil. This new species clearly belongs to *Martinella* based on its prophylls, the continuous interpetiolar ridges and the corolla shape. However, *Martinella insignis* can be distinguished by the membranous leaflets, 5-lobed aristate calyces, and yellow corollas. In addition, *Martinella insignis* also has pocket-shaped leaf domatia (Fig. 2C) and a puberulous indument of glandular stipitate trichomes that covers almost all organs, with variable density (Fig. 2E–F). These trichomes may also be found in *Martinella obovata* and a few other species in Bignonieae, and are typically formed by a multicellular secretory head, supported by a uniseriate stalk (Nogueira et al 2013). Only corollas and fruits lack these; the corollas are glabrous, and fruits have simple, deciduous trichomes (Fig. 2G). Similar to the other species, the root system of *Martinella insignis* has unusual tuberous portions (Fig. 2D) that might represent an adaptation to the sandy soils, by accumulating water. However, the anatomical structure and function of these are yet unknown. Alwyn Gentry had already noted this new taxon, and had proposed the epithet “insignis” in sched.; his earlier findings are here accredited.

#### Additional collections examined.

**Brazil.** Bahia: Guaratinga, Fazenda Vitória, 16°43’S, 39°46’W, 29 October 1979, *L.A. Mattos Silva & H.S. Brito 634* (CEPEC, MO). Itabuna, Alcobaça para(ramal) S. Antonio, 24 *January* 1972, *R.S. Pinheiro 1759* (CEPEC, MO). Itamaraju, *S. Mori, L.A. Mattos Silva & T.S. Santos 10743* (CEPEC, MO), Itamaraju, Fazenda Riacho das Pedras, prop. Sr. Gersino Antônio Bronzon, 17°08'48"S, 39°21'53"W, 12 February 2007, *R.A.X. Borges, A. Amorim, W.W. Thomas, L.C. Gomes, S. Sant’Ana & O. Cruz 825* (CEPEC, SPF). Espírito Santo: Linhares, Reserva Natural da Companhia Vale do Rio Doce (“Reserva de Linhares”), MME, 19°07'57.5"S, 40°04'06.3"W, 65m, 14 December 2007, *A.R. Zuntini, W.A.A. Pires & G.S. Siqueira 151* (CVRD, RB, SPF), *A.R. Zuntini, E. Françoso, J. Lopes & V. Augusto 321* (SPF). Governador Lindenberg, Pedra de Santa Luzia, 420–590 m, 7 November 2007, *V. Demuner, T.A. Cruz & R.R. Vervloet 4481* (MBML, SPF). Sooretama, Mata de tabuleiro situada ao Noroeste da sede da Reserva da Sooretama, 14 July 1969, *D. Sucre 5519* (RB–photo).

### 2. Martinella iquitoensis

A. Samp., Ann. Acad. Bras. Sci. 7: 122. 1935.

http://species-id.net/wiki/Martinella_iquitoensis

Martinella manaosiana A.Samp., Bol. Mus. Nac. Rio de Janeiro 12(3, 4): 84. 1936. TYPE: BRAZIL. Amazonas: Manaus, 25 July 1931, *A. Ducke sn* (holoype: RB-24095!; isotype MO-2193049!, RB-24095 [second sheet]!)

#### Type.

PERU. Loreto: Iquitos, 23 February 1924, *J.G. Kuhlmann 1492* (holotype RB-22027!; isotypes, MO-2192060!, RB-22027 [second sheet]!).

#### Distribution and habitat.

This species is distributed widely in the Amazon basin (Brazil, Colombia, Ecuador, Peru and Venezuela), typically in sandy soils (Lohmann and Taylor 2014; Fig. 3).

#### Conservation status.

*Martinella iquitoensis* is distributed geographically through an area that is < 2000 km^2^, with seven Rapoport (1982) sub-populations known to date and ≥ 20% of its known individuals occurring outside Protected Areas, making it susceptible to the current reduction and degradation of its habitat. Therefore, this species is here considered as Vulnerable [VU B2ab(ii,iii)] according to the IUCN criteria (IUCN 2012; IUCN Standards and Petitions Subcommittee 2014).

#### Discussion.

This species was distinguished by Sampaio from *Martinella obovata* based on the corolla color and size, leaflet texture and size, tendril type, and calyx indument. However these characters have proven to be fairly variable, especially in *Martinella obovata*, leading to morphological overlap between those taxa. The lack of a clear morphological discontinuity combined with the sympatric distributions, make these species hard to separate. Moreover, the difficulty in delimitating these two species can be observed in the few treatments that dealt with those species, which is particularly evident in the contrasting species keys presented (Sampaio 1935; MacBride 1961; Gentry 1982; Gentry 2009).

A character that might help telling these species apart is the inflorescence structure: a thyrse or panicle in *Martinella iquitoensis* versus a raceme in *Martinella obovata*. This character, combined with leaflet base, is here proposed as diagnostic for each species; however, the examination of additional material is necessary to validate its usefulness and consistency.

### 3. Martinella obovata

(Kunth) Bureau & K.Schum., in Mart., Fl. Bras. 8(2): 161, tab. 84. 1896.

http://species-id.net/wiki/Martinella_obovata

Spathodea obovata Kunth, Nov. Gen. Sp. (quarto ed.) 3: 147. 1818. [1819].
Bignonia obovata (Kunth) Spreng., Syst. Veg. 2: 830. 1825.
Macfadyena obovata (Kunth) Miers, Proc. Roy. Hort. Soc. London 3: 200. 1863.

#### Type.

COLOMBIA. Turbaco, s.d., *Humboldt and Bonpland 1391* (holotype, P-Bonpl. [P00670823]!)

#### Distribution and habitat.

This species is found from Central America and the Antilles through northern South America to the southern Amazon basin, in the Antilles, Belize, Bolivia, Brazil, Colombia, Costa Rica, Ecuador, French Guiana, Guatemala, Guyana, Honduras, Mexico, Panama, Peru, Surinam, and Venezuela, in different soil types and habitats (Lohmann and Taylor 2014; Fig. 3).

#### Conservation status.

*Martinella obovata* is distributed geographically through an area that is ≥ 2000 km^2^, with < 20% of its known individuals occurring outside Protected Areas, and seventy-two Rapoport (1982) sub-populations known to date. Therefore, this species is here considered as Least Concern [LC] according to the IUCN criteria (IUCN 2012; IUCN Standards and Petitions Subcommittee 2014).

#### Discussion.

This species is the most variable and has the largest distribution in the genus (Central America and Caribbean throughout Amazon basin). Such variation is product of its phenotypic plasticity and wide ecological range, and is responsible for making this species hard to be distinguished from *Martinella iquitoensis* (as discussed above). Nonetheless, this high variation may also be seen as an evidence of a species complex, in which case, additional studies would be needed. For a complete list of synonyms see Gentry (1977).

## Supplementary Material

XML Treatment for Martinella

XML Treatment for Martinella insignis

XML Treatment for Martinella iquitoensis

XML Treatment for Martinella obovata
